# Recent advances of natural and bioengineered extracellular vesicles and their application in vascular regeneration

**DOI:** 10.1093/rb/rbac064

**Published:** 2022-09-05

**Authors:** Jianxiong Xu, Jinxuan Wang, Yidan Chen, Yuanfang Hou, Jianjun Hu, Guixue Wang

**Affiliations:** Key Laboratory for Biorheological Science and Technology of Ministry of Education, State and Local Joint Engineering Laboratory for Vascular Implants, Bioengineering Modern Life Science Experiment Teaching Center of Bioengineering College, Chongqing University, Chongqing 400030, China; Key Laboratory for Biorheological Science and Technology of Ministry of Education, State and Local Joint Engineering Laboratory for Vascular Implants, Bioengineering Modern Life Science Experiment Teaching Center of Bioengineering College, Chongqing University, Chongqing 400030, China; Key Laboratory for Biorheological Science and Technology of Ministry of Education, State and Local Joint Engineering Laboratory for Vascular Implants, Bioengineering Modern Life Science Experiment Teaching Center of Bioengineering College, Chongqing University, Chongqing 400030, China; Key Laboratory for Biorheological Science and Technology of Ministry of Education, State and Local Joint Engineering Laboratory for Vascular Implants, Bioengineering Modern Life Science Experiment Teaching Center of Bioengineering College, Chongqing University, Chongqing 400030, China; Department of Pathology, Guizhou Provincial People’s Hospital, Guiyang 550002, China; Key Laboratory for Biorheological Science and Technology of Ministry of Education, State and Local Joint Engineering Laboratory for Vascular Implants, Bioengineering Modern Life Science Experiment Teaching Center of Bioengineering College, Chongqing University, Chongqing 400030, China

**Keywords:** cardiovascular disease, extracellular vesicle, vascular regeneration, biomedical engineering

## Abstract

The progression of cardiovascular diseases such as atherosclerosis and myocardial infarction leads to serious vascular injury, highlighting the urgent need for targeted regenerative therapy. Extracellular vesicles (EVs) composed of a lipid bilayer containing nuclear and cytosolic materials are relevant to the progression of cardiovascular diseases. Moreover, EVs can deliver bioactive cargo in pathological cardiovascular and regulate the biological function of recipient cells, such as inflammation, proliferation, angiogenesis and polarization. However, because the targeting and bioactivity of natural EVs are subject to several limitations, bioengineered EVs have achieved wide advancements in biomedicine. Bioengineered EVs involve three main ways to acquire including (i) modification of the EVs after isolation; (ii) modification of producer cells before EVs’ isolation; (iii) synthesize EVs using natural or modified cell membranes, and encapsulating drugs or bioactive molecules into EVs. In this review, we first summarize the cardiovascular injury-related disease and describe the role of different cells and EVs in vascular regeneration. We also discuss the application of bioengineered EVs from different producer cells to cardiovascular diseases. Finally, we summarize the surface modification on EVs which can specifically target abnormal cells in injured vascular.

## Graphical Abstract

**Figure:**
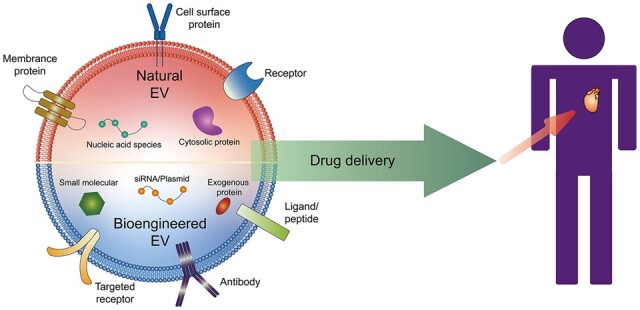

## Introduction

Cardiovascular injury is the result of many diseases, such as atherosclerosis, percutaneous coronary intervention, autologous saphenous vein coronary artery bypass grafting and ischemia–reperfusion (I/R). Vascular regeneration or reconstruction after injury is critical for functional recovery. The main problems after vascular injury are endothelial inflammation and thrombosis, which delay the intimal formation and accelerate intimal hyperplasia [1–4]. Conventional clinical treatments are anti-inflammation, anti-thrombus and induction of intima formation [4–6]. Due to the low targeting of drugs and adverse drug reactions such as bleeding events and restenosis, it is difficult for repairing vascular injury with desired effect.

Extracellular vesicles (EVs), which are cell-derived membrane vesicles, are originally discovered to be the mediators of intercellular communication. EVs are natural carriers produced by various cell types, carrying lipids, proteins and RNA [7]. In general, EVs are categorized into four classes: exosomes, 40–100 nm; membrane particles, 50–80 nm; microvesicles, 100–1000 nm; apoptotic vesicles, more than 800 nm [8, 9]. Meanwhile, the small size of EVs (<200 nm), which can penetrate the endothelial barrier and enter deep tissue, are widely applied in nanomedicine [10]. Studies have shown that natural EVs play an important role in injured vascular regeneration through regulating vascular smooth muscle cells (VSMCs) phenotype transition and endothelial cells (ECs) integrity [11]. However, the complex extraction process and biological function of EVs limit their application in clinical treatment. Bioengineered EVs derived from cell membranes, which have both original and modified functions, are widely used in targeted therapy. In recent years, bioengineered EVs have been used as carriers for nanomaterials, which have advantages in low cytotoxicity, strong targeting and immune escape [9].

In this review, we briefly introduce the molecular mechanism and regenerative process of vascular injury. We summarize the application of natural EVs and bioengineered EVs in the treatment of cardiovascular diseases and describe the function of EVs in injured vascular regeneration. Meanwhile, we further compare the characteristics and functions of different EVs and summarize targeted strategies for vascular injury-related diseases. We also introduce bioengineered methods to construct EVs and improve the bioactivity and targeting of EVs. Finally, we discuss the application of bioengineered EVs in the therapy of vascular injury-related diseases and the future of bioengineered EVs.

## The molecular mechanism of vascular regeneration after injury

**Figure 1.**
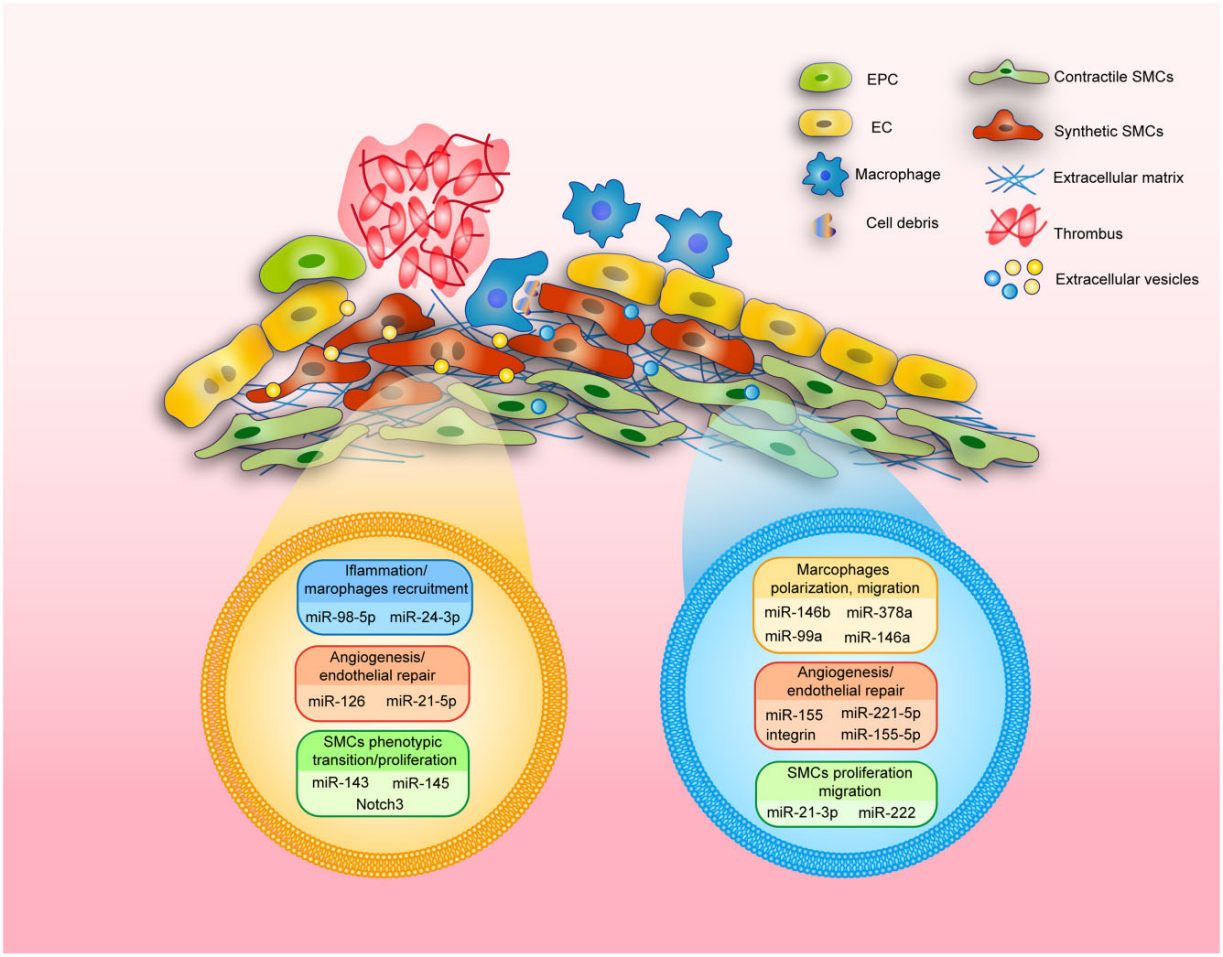
The functions of extracellular vesicles (EVs) in vascular injured disease. The injury repair of cardiovascular is a complex process, which include thrombosis, the adhesion and infiltration of macrophages to the activated ECs, uptake of apoptotic cells and debris, the directed migration, proliferation and phenotype transition of VSMCs, the recruitment and migration of endothelial progenitor cells (EPCs) and ECs, and the synthesis of extracellular matrix to repair the injured sites. EVs are mainly released from macrophages and ECs/EPCs, which contain variety of microRNA (miRNA) and proteins to modulate cell function and microenvironment such as: inflammation (miR-98-5p, miR-24-3p) [14, 15], angiogenesis (miR-126, miR-21-5p, miR-155, miR-221-5p, integrin, miR-155-5p) [16–20], SMC phenotypic transition (miR-143, miR-145, Notch) [21, 22], macrophages polarization (miR-146b, miR-378a, miR-99a, miR-146a) [23, 24] and SMC proliferation/migration (miR-21-3p, miR-222) [25, 26].

Vascular injury, caused by blood flow shear stress, inflammation and fibrosis, is the pathological foundation and causes of multi-cardiovascular diseases. Meanwhile, vascular injury usually occurs after the surgery of arterial occlusive disease, such as carotid endarterectomy, balloon dilatation surgeries and so on [12, 13]. The regeneration of injured vascular is a complex but orderly process. Generally, there are three stages of vascular repair, including inflammation, neointima formation and vascular remodeling. During the repair process, the first step is platelet aggregation and inflammatory cell infiltration; second, VSMCs will proliferate and ECs migrate to the vascular injury site; finally, the extracellular matrix is deposited in the injured site and then vascular remodeling has been completed (Fig. 1).

### ECs and endothelial progenitor cells

ECs, as the interface between the blood and tissues, are important in anti-thrombus, anti-inflammation and vascular barrier function. Importantly, ECs, which play an important role in intracellular communication during vascular injury, are considered as potential mediators of cardiovascular diseases. Furchgott and Zawadzki have first demonstrated that ECs can release endothelium-derived relaxing factors [3], which participate in multi-biological processes, such as oxidative stress, inflammation, vasoconstriction and thrombosis [27, 28].

After intimal injury, the repair of endothelial layer includes two main ways [29]. First, the regenerative cells at injured sites are mostly derived from nearby uninjured cells. Researchers find that the re-endothelialization of arterial intima has three independent stages: cleaning, rapid re-endothelialization/proliferation and maturation. This dynamic process starts at 6–24 h after endothelial injury. Then, ECs are able to complete re-endothelialization at 48 h after injury, and maturation when the ECs form continuous and integrity intimal structure at 96 h [30, 31]. Second, the circulating EPCs, including early EPCs and late EPCs, can promote the regenerative process of vascular intima [32, 33]. Early EPCs secrete a variety of angiogenic factors such as vascular endothelial growth factor (VEGF), stromal cell-derived factor 1 and so on, which participate in recruiting mature ECs and regulating ECs proliferation during vascular injury [34–36]. Late EPCs exhibit high cell proliferation rate and strong ability to promote regeneration [37, 38]. Wakabayashi *et al*. have reported that CD157^+^ EPCs display regenerative property and are essential for re-endothelialization [39]. Meanwhile, EPC/EC-derived EVs, containing varieties of miRNA and protein, also play a crucial role in attenuating vascular injury and promoting vascular regeneration [40, 41] (Fig. 1). During vascular injury, endothelial-to-mesenchymal transition (EndMT), as a cellular differentiation process, results in migration and fate transition of EC. The EndMT-derived myofibroblasts not only attenuate inflammation but also have the ability to deal with dead cells, which is important for myocardial infarction (MI) recovery [42]. However, excessive EndMT will affect collagen–matrix metalloproteinase balance, fibrosis and correlate with an unstable plaque phenotype in atherosclerosis and in-stent stenosis [43, 44]. Thus, the molecular mechanism of endothelial phenotype transition during vascular injury provides an insight into targeted therapy for re-endothelialization and the treatment of vascular disorders.

### Vascular smooth muscle cells

VSMCs, as the media parts of vascular wall, are essential for vascular homeostasis. During vascular injury, VSMCs will switch cell fate into migratory and proliferative phenotypes. Therefore, VSMCs can be diverted into ‘contractile’ type (differentiated, non-proliferative) and ‘synthetic’ types (dedifferentiated, proliferative) [45]. The ‘synthetic’ VSMCs have stem cell properties, which exhibit high proliferation and soft stiffness, in response to inflammation and biomechanics during vascular injury [46, 47]. Meanwhile, the unexpected activated VSMCs will migrate from media to intima and start to proliferate after excessive vascular injury [48]. These alterations will result in intimal hyperplasia and lead to vascular stenosis, which significantly increases the risk of cardiovascular diseases, such as MI and stroke. Many growth factors and inflammatory factors, such as transforming growth factor-β, platelet-derived growth factor and interferon, lead to transforming the phenotype of VSMCs and regulating VSMCs proliferation [49]. Furthermore, researchers have also reported that EVs secreted by multiple cell types from pathological microenvironment play an important role in VSMCs phenotype transition and vascular tissue repair [11, 50, 51] (Fig. 1). Thus, EVs loading different contents may effectively target abnormal VSMCs and have potential in the therapy of cardiovascular disease.

### Macrophages

Macrophages, as the important member of immune cells, can be recruited to the injured sites within 15 min to resolve inflammation and promote tissue repair [52–54]. In vascular injured diseases, such as atherosclerosis, MI and stroke, macrophages are essential for vascular repair and regeneration, contributing to ECs migration and proliferation, neovascularization and SMCs phenotype transition [55–57]. During vascular repair, macrophages will differentiate into pro-inflammatory M1 and anti-inflammatory M2, which is according to the different processes of injury [58]. M1 macrophages can secrete inflammatory cytokines to promote the initial infiltration of circulatory leukocytes [59]. After the early inflammatory phase, macrophages will clean necrotic cell debris via phagocytosis and conversion of M1 to pro-repair and anti-inflammatory M2 phenotype [60]. The M2 macrophages can promote cellular proliferation and vascular remodeling by controlling the production of growth factors, such as VEGF, transforming growth factor β1, insulin-like growth factor 1 and so on [55]. Furthermore, Liu *et al*. have reported that macrophages also directly mediate vascular repair via using mechanical forces to drag ECs healing and facilitate tight junction [61].

EVs from macrophages also play an important role in regulating vascular microenvironment and various pathophysiological processes. Recent research revealed that macrophages-derived EVs can regulate vascular ECs proliferation, migration and angiogenesis in cardiovascular disease [62] (Fig. 1). Thus, EVs as the regulator of cell/cell communication may act as targeting liposomes for drug delivery and therapy during vascular injury.

## The different derived EVs for vascular therapy

**Figure 2.**
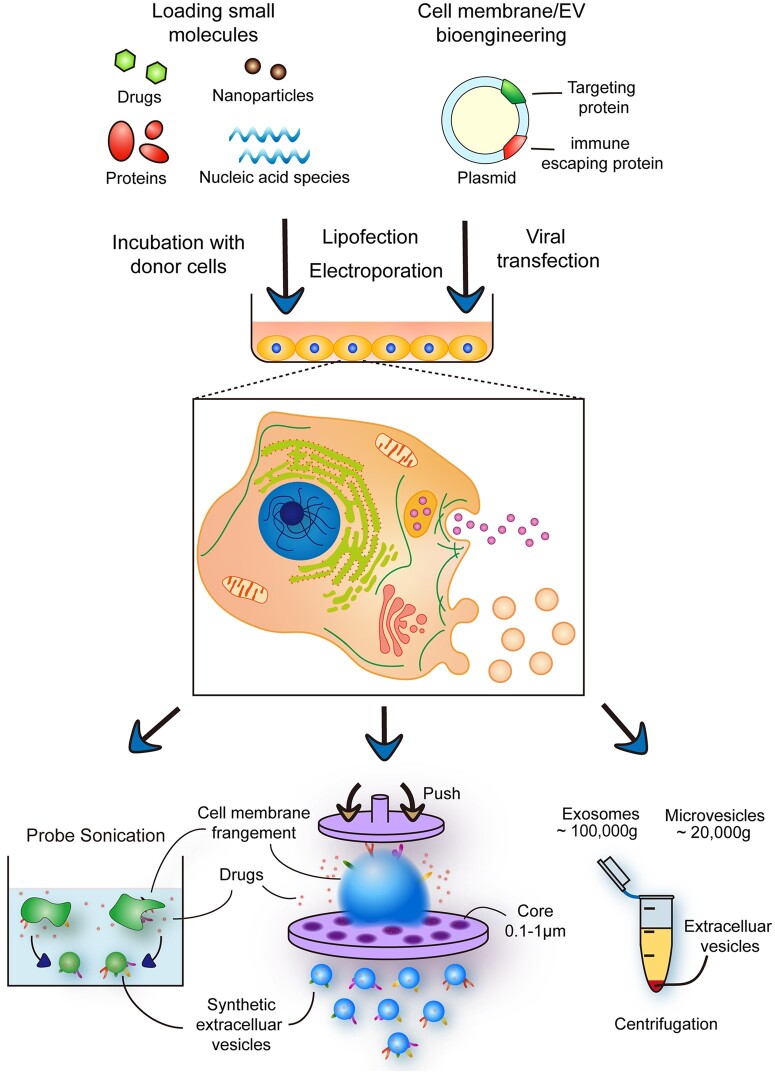
The synthetic strategies of bioengineered EVs. Producer cells can be loaded with therapeutic molecules such as drugs, nanoparticles (NPs), nucleic acid species and proteins via incubation with cells, lipofection and electroporation. Producer cells also can be transfected with plasmid or mRNA via lipofection, electroporation and viral transfection to express protein and peptides which have therapeutic and targeting characteristics. EVs from producer cells are then isolated through centrifugation with different centrifugal force. Moreover, EVs also can be synthesized by membrane extrusion and probe sonication. The bioengineered cell membrane fragments are extruded through pore size 0.1–1 µm polycarbonate film or sonicated in a water bath to synthesize biomimetic EVs. Biomimetic EVs have the biological inherencies from the producer cells including specifically surface receptor and bioactive molecular. Moreover, the drugs can be loaded in biomimetic EVs during synthesis.

In recent years, nanotechnology has been widely used in the diagnosis and treatment of cardiovascular diseases [63, 64]. However, it is difficult for synthetic nano-drugs to target vascular injured sites through cell biological interactions. EVs are cell-derived membrane vesicles, which are essential for intracellular biological transduction and cellular communication [65]. In vascular injury sites, EVs released from activated vascular cells containing multi-bioactive factors are able to become promising candidates to treat cardiovascular diseases, which have ability in regulating cell proliferation, migration, trans-differentiation and apoptosis (Table 1). Meanwhile, because almost all cells are capable of secreting EVs, EVs are present in various organizations with long-time circulation and can be artificially isolated from body fluids such as blood, urine, cerebrospinal fluid and saliva in large quantities [66]. Therefore, using EVs as the shell of nano-drugs can prolong circulation time and greatly improve delivery efficiency. Moreover, researchers have created bioengineered EVs via modifying EV membranes and loading drugs into EVs to improve bio-targeting and bioactivity (Fig. 2) [67–69]. Furthermore, biomimetic EVs system constructed by cell membrane retains the membrane surface structure and unique biological functions. These systems have obvious advantages for systematic delivery of drugs in cardiovascular diseases, e.g. membrane encapsulating drugs at nanoscale can protect them from immune system and increase drug delivery efficiency with low toxicity [13, 70]. Due to different cell-derived EVs playing distinct roles in the process of vascular regeneration, it is important to select suitable EV systems for the therapy of vascular injured diseases. Here, we summarize the commonly used EVs system in vascular injured diseases including red blood cell (RBC)-derived EVs, EC-derived EVs, macrophage-derived EVs, platelet-derived EVs and stem cell-derived EVs (Table 1).

**Table 1.** Natural and bioengineered EVs for the treatment of vascular injured diseases

Donor cell	Methods	Animal model	Administration	Characteristics	Effects	References
RBC	Extrusion through 200 nm ppm Loading with PLGA–rapamycin NPs	Mouse atherosclerosis	i.v.	Immune escape Long circulating time	Decrease the size of atherosclerotic plaque Reduces inflammation	[71]
	Probe sonication Modification with stroke homing peptide Loading with PHB-dextran -NR2B9C NPs	Mouse I/R	i.v.	Targeting stroke area ROS-responsive drug release High biocompatibility	Decreases infarct size Prevent brain neurons	[72]
	Probe sonication Modifying with fibrin-targeting peptide loading with dextran–tirofiban conjugate NPs	Mouse thrombosis	i.v.	Targeting fibrin ROS-responsive drug release high biocompatibility	Increases anti-thrombotic activity	[73]
EC	Centrifugation at 20 500 g	Mouse atherosclerosis	i.v.	miR-143/145 enrichment	Decrease the size of atherosclerotic plaque Transits SMC to athero-protective phenotype	[21]
	Overexpression Klf2 in donor cells Gradient centrifugation	Mouse I/R	i.v.	miR-24-3p enrichment	Decrease I/R injury Reduces the recruitment of Ly6C^+^ monocyte	[15]
	Overexpression Cxcr4 in donor cells Extrusion through 200 nm ppm Loading with HOP conjunct rapamycin NPs	Mouse I/R	i.v.	Targeting Sdf-1 high expressed area ROS-responsive drug release	Decreases infarct size Reduces radical-induced damage and inflammation	[74]
Macrophage	IL-4 stimulates donor cell Centrifugation at 100 000 g	Mouse atherosclerosis	i.p.	miRNA-99a/146b/378a enrichment	Decreases inflammation and necrotic lesion areas	[23]
	Centrifugation at 100 000 g	Rat stent implantation	Local delivery	Using M2 macrophage-derived exosomes	Accelerates vascular tissue repair Promotes VSMC dedifferentiation	[11]
	Centrifugation at 100 000 g Loading with HAL by electroporation	Mouse atherosclerosis	i.p.	Targeting chemokine-enriched area	Increases the anti-inflammation effects Alleviates atherosclerosis	[75]
	IL-1βR, IL-6R and TNF-αR plasmids are transfected in donor cells Extrusion through 400 nm ppm Loading with miR-199a-3p-PEG–PLA NPs	Mouse MI	i.v.	Targeting IL-1β, IL-1β, TNF-α enriched area miR-199a-3p enrichment	Accelerates the recovery of cardiac function Prevents hypoxia‐induced apoptosis	[76]
	Extrusion and sonication Loading with Oxi-COS-atorvastatin NPs	Mouse atherosclerosis	i.v.	ROS-responsive drug release	Decrease the size of atherosclerotic lesion Suppress local inflammation and ROS	[77]
Platelet	Centrifugation at 100 000 g	Rat MI	Left ventricle	Stimulates VEGF, bFGF signaling pathway	Improve the process of revascularization	[78]
	Probe sonication Loading with docetaxel–PLA NPs	Balloon vascular injury	i.v.	Collagen binding (injured target) Immuno-compatibility	Suppresses coronary restenosis	[79]
	Modifying with PEG on donor cell Probe sonication Loading with PLGA–rapamycin NPs	Mouse atherosclerosis	i.v.	Accumulation in atherosclerotic plaque	Attenuates the progression of atherosclerosis	[80]
Progenitor/stem cell	Centrifugation at 100 000 g	Rat stent implantation	Coating on stent	Stem cell-derived EVs have pro-healing property	Accelerates re-endothelialization	[81]
	Overexpression Gata4 in donor cell 0.2µm filtration and gradient certification	Rat MI	i.o.	Enrich anti-apoptotic miRNAs (miR-19a)	Restores cardiac contractile function Reduces infarct size	[82]
	Overexpression Cxcr4 in donor cell Probe sonication Loading with VEGF–PLGA NPs by sonication	Mouse hindlimb ischemic	i.v.	Targeting ischemic tissue	Enhances blood reperfusion Accelerates limb salvage	[83]
	Loading iron oxide NPs in donor cell Extrusion through 400 nm ppm	Rat I/R	i.v.	Therapeutic growth factors Magnetically guided, targeted drug delivery	Decreases infarction volume Promotes angiogenesis, anti-apoptosis and anti-inflammation	[84]

ppm, polycarbonate porous membrane; PEG-PLA, poly (ethylene glycol–polylactic acid); PLGA, poly (lactic-co-glycolic acid); HOP, p-hydroxybenzyl alcohol-oxalyl chloride-poly (ethylene glycol); Oxi-COS, amphiphilic oxidation-sensitive chitosan oligosaccharide; HAL, hexyl 5-aminolevulinate hydrochloride; i.v., intravenous injection; i.p., intraperitoneal injection; i.o., intramyocardial injection; local delivery, drugs were preloaded into pluronic gel F-127 (Sigma) and locally dress around the injured artery; I/R, ischemia-reperfusion; MI, myocardial infarction; NPs, nanoparticles.

### RBC-based EV system

RBCs (also as erythrocyte), as the most abundant type of blood cells, play an important role in transporting oxygen and nutrients [85]. Meanwhile, RBCs not only have long-term circulatory property, but also RBCs-derived microvesicles can regulate a variety of cardiovascular diseases [86]. These long-circulating, biocompatible and non-immunogenic properties enable RBCs and their derived EVs to serve as protective shells for cargoes [87]. Recently, Usman *et al*. constructed a RBCs-derived EVs delivery system to load miRNA and CRISPR–Cas9 system showing higher transfecting efficiency than traditional reagents [88]. Furthermore, RBC membrane also has self-immune escape marker Cd47 and polysaccharide coating, which are essential for cell stability and immune escape [89]. Thus, RBCs membrane can be utilized to package drugs or small molecular, such as polymeric NPs and other nano-drug to construct biomimetic EVs with extended circulation time up to 72 h [90]. Our lab recent work uses RBCs encapsulating core-shell structured nanocomplexes to target atherosclerosis. Animal experimental results show that RBC-derived biomimetic EVs reduce immune clearance and increase the accumulation of NPs in atherosclerotic plaque [71]. Moreover, due to its highly flexible structure, RBC-derived biomimetic EVs can load different core NPs including polymeric NPs [91], magnetic NPs [92], gold NPs [93] and so on. These excellent properties make RBCs-based biomimetic EVs system have great potential in the treatment of cardiovascular diseases (Table 1).

### EC-based EV system

ECs, as the interface between blood and vascular wall, are considered as the functional regulator in vascular homeostasis, especially in barrier function, inflammation and thrombus. Recent study has reported that EC-derived small EVs (sEVs) act as a new link between ECs and SMCs in vascular and play an important role in angiogenesis and the transition of SMC phenotype [94]. EVs containing miR-143/145 that are isolated from Klf2-overexpressing ECs can regulate SMC phenotypes and reduce atherosclerotic lesion formation [21]. Moreover, these KLF2-overexpressing EC-derived EVs also can attenuate myocardial I/R injury and reduce the recruitment of Ly6C^+^ monocyte [15]. Due to EC-derived EVs having great potential in cardiovascular disease, our lab recent work uses CXCR4-overexpressing primary mouse thoracic aorta EC-derived EVs loading with HBA-PC-PEG conjunct rapamycin NPs to target SDF1 highly expressed and ROS accumulated area during cerebral I/R injury. These synthetic EVs significantly improve cerebral I/R injury and suppress local inflammation [74]. Thus ECs-based EVs have been widely applied in the treatment of cardiovascular diseases.

### Macrophage/monocyte-based EV system

Macrophage, which has specific receptors to recognize inflammation, tissue debris and foreign invasion, is the ‘guardian’ of the body [95]. Because EVs can inherit the membrane functional receptors from donor cells, macrophage-derived EVs are widely applied in injury and inflammation-related diseases [96, 97]. Macrophage-derived EVs show perfect inflammation-tropism and anti-inflammation properties via high expression of chemokine receptors and releasing anti-inflammatory cytokines [98]. Moreover, because M2 macrophage and its EV play an important role in targeting and resolving inflammation and tissue remodeling, researchers have utilized M2 macrophage-derived EVs to promote vascular regeneration. Recently, researchers have reported that bioengineered M2 macrophages exosomes loading with hexyl 5-aminolevulinate hydrochloride via electroporation exhibit excellent abilities for targeting chemokine-enriched area and reducing vascular necrotic area in atherosclerosis [75]. Furthermore, because EVs can involve in cell-to-cell communication, they contain numerous cargoes including miRNAs, lncRNAs, proteins and lipids that may act as therapeutic agents in cardiovascular diseases (Fig. 1) [99]. Bouchareychas *et al*. utilize IL-4 to foster macrophages to M2 polarization and find that these macrophage-derived exosomes, which contain miRNA-99a/146b/378a, can reduce inflammation and atherosclerotic plaque in vascular injury [23]. Because macrophages can adhere on vascular injured site, macrophage membrane is utilized to encapsulate ROS-responsive PCM-rapamycin NPs for targeting ROS accumulated and inflammatory area in pathological vascular injury. These biomimetic EVs can effectively inhibit intimal hyperplasia with low cytotoxicity on ECs [97, 100]. Moreover, Martinez *et al*. have utilized macrophage membrane proteins hybridizing with liposomes to target inflammatory ECs [101]. Because macrophage-based EV can target inflammatory site, which exhibits superior accumulation in injured area and can load a wide variety of solubility nano-drugs and contrast agents, they are increasingly used in all aspects of vascular injury-related disease therapy (Table 1).

### Platelet-based EV system

Platelets are non-nucleated blood cells produced by mature megakaryocytes in the bone marrow and lungs, which play an important role in the process of coagulation, hemostasis, inflammation and immune regulation [102, 103]. Platelet-derived EVs contain nucleic acids, growth factors, lipids and proteins, can regulate injury repair, inflammation and immune response after vascular injury [104]. Brill *et al*. have reported that platelet-derived EVs can stimulate angiogenesis through VEGF signaling after MI [78]. Moreover, EVs derived from thrombin/collagen-induced platelets can enhance the adhesion of early outgrowth cells in vascular injured sites and promote re-endothelialization [105]. Meanwhile, due to platelets having the ability to adhere and aggregate in injured site, Hu *et al*. prepare biomimetic EVs by encapsulating docetaxel-loaded poly(lactic-co-glycolic acid) (PLGA) NPs in platelet membrane. These biomimetic EVs have great immuno-compatibility and collagen targeting, which can selectively adhere to injured vascular and efficiently suppress coronary restenosis [79]. Moreover, to improve the circulation time of EVs in blood, PEG was utilized to modify on platelet membrane. These PEG-modified platelet-derived EVs loading with PLGA–rapamycin NPs exhibit 4.98-fold accumulated efficiency than normal NP in atherosclerotic arterial trees and significantly attenuate the progression of atherosclerosis [80]. Platelet-derived EVs preferentially accumulate in the collagen exposure area, which is associated with intimal injury, demonstrating an effectively targeted ability to vascular injured sites.

### Progenitor/stem cell-based EV system

Stem/progenitor cells are self-renewing, multipotent cells that reside in various tissues and play an important role in tissue repair, anti-inflammation and multilineage differentiation [106, 107]. Mesenchymal stem cell (MSC) as the abundant stem cell in vascular, which can produce amounts of EVs during physiological environment, is essential for tissue repair and differentiating to replace injured cells [108]. Meanwhile, a large amount of miRNA has been identified in MSC-derived EVs, many of which can inhibit inflammation and promote neovascularization [82]. Thus, recent studies have used MSC-derived exosomes to treat cardiovascular injury. Hu *et al*. fabricate MSC-derived exosome-released stent that can promote vascular healing and accelerate re-endothelialization after stent implantation [81]. Moreover, Zhao *et al*. find that MSC-derived exosomes can restore cardiac contractile function and transform macrophages to M2 phenotype through miR-182 [109]. Furthermore, peptide GSPREYTSYMPH is used to cross-link with MSC-derived nanovesicles, which can target disturbed flow site and significantly contribute to endothelial recovery after injury [110]. Due to the targeting ability of natural EVs still needing to enhance, CXCR4 is overexpressed in stem cells through plasmid transfection to acquire robust targeting. Then PLGA–VEGF NPs are encapsulated by cell membrane to construct nanovesicles, which have great potential for delivering VEGF to injured sites and promoting the recovery of vascular function [83]. Furthermore, Kim *et al*. co-cultured MSCs with iron oxide NPs to stimulate the expression of therapeutic growth factors in MSC and made MSC-derived EVs obtain magnetic targeting. The magnetic navigation makes MSC-derived EVs localize on the ischemic lesion and have great effects on the treatment of ischemic stroke [84]. Stem cell-derived EVs have great potential in the treatment of vascular injury due to their regenerative cargos.

### Hybrid membrane-based biomimetic EV system

Cell membrane-wrapped biomimetic EV, which can be simply fabricated, is a new targeted nano-drug delivery system. Although single-type cell membrane-based EVs system has been applicated in cardiovascular disease therapy, it is still struggle to have therapeutic efficacy in the pathological microenvironment after vascular injury. Thus, recent researchers develop hybrid RBC–EC membrane-cloaked NPs to target choroidal neovascularization, in which RBC membrane is used to reduce immune phagocytosis and EC membrane is used as anti-VEGF nano agents to target retinal endotheliocytes [111]. Moreover, because RBCs have long-term circulatory property, RBC–platelet hybrid and RBC–cancer membrane hybrid are used to target tumor area and prolong drug circulation time [112–114]. Meanwhile, due to the platelet membrane also having immune-evading and cancer cell-binding ability, platelet membranes are widely used to fuse with other cell membranes such as leukocyte and cancer stem cell [115–117]. Furthermore, platelet membrane also is used to fuse with stem cell exosomes to enhance exosomes’ capability to target injured vascular [118]. Combination of the differently biological membrane can obtain both functions and characteristics of producer cells, which improve the targeting ability and circulating time.

## The bioengineered strategies for EVs to target vascular injured site

EVs have been considered as advanced strategies to treat vascular injured disease, whereas, systemic exposure and off-target effect have limited the development of cardiovascular therapies. Moreover, the repair of vascular injury, as a multiple physiological process, is regulated by a variety of cells and factors, which make drugs hardly direct to the diseased site and have therapeutic index. For example, after stent implanting into the carotid arteries, preventing neointimal hyperplasia and re-endothelialization are the most important events to avoid arterial re-obstruction. Although anti-proliferative drugs such as rapamycin or paclitaxel have great effect on preventing neointimal thickening, the re-endothelialization also will be delayed by the suppression of endothelial proliferation. Meanwhile, compared with paclitaxel-eluting stents, MSC-derived exosomes-eluting stent accelerates re-endothelialization, but also promotes SMC proliferation in vascular injured sites [81]. Thus, it is important to modify EVs with targeting proteins or peptides that can specifically bind with cellular receptors and extracellular components expressed in injured vascular and make EVs-based systems can efficiently deliver drugs to targeted sites or even directly to the diseased/abnormal cells. Here, we summarize the targeting peptides and proteins for cardiovascular diseases (Table 2).

**Table 2.** Potential strategies for targeting abnormal cells in cardiovascular diseases

Targeting cell	Targeting point	Disease	Targeting agent/peptide	Effects	References
EC	VCAM1	Atherosclerosis	VHPK (peptide)	Target inflammatory EC and reduce atherosclerotic plaque	[119]
		Atherosclerosis	Integrin α_4_β_1_ (protein)	Target activated EC and reduce atherosclerotic plaque	[70]
	E-selectin	Atherosclerosis	HPMA (polymer)	Target inflammatory EC and reduce vascular inflammation	[120]
	α-2Ars	Atherosclerosis	Cys-L9R-Cys (peptide)	Target lipid-activated EC and enhance eNOS expression	[121]
Macrophage	CCR2	Atherosclerosis	YNFTNRKISVQRLASYRRITSSK (peptide)	Target monocyte with inflammatory response imaging atherosclerotic area	[122]
	CCR5	Vascular injury	DAPTA (peptide)	Target recruited monocytes and imaging vascular injury	[123]
	P32	Atherosclerosis	LYP-1 (peptide)	Target macrophages in atherosclerotic plaque	[124]
	CD36	Atherosclerosis	KOdiA-PC (lipid)	Target macrophages in atherosclerotic plaque	[125]
SMC	PDGFRβ	Vascular injury	PDGF-BB peptide	Reduce restenosis and neointimal hyperplasia.	[126]
	Heparan sulfate	Hypertension	CAR (peptide)	Inhibit SMC proliferation and migration	[127]
Cardiomyocytes	Unknown	MI	CSTSMLKAC (peptide)	Reduce cardiomyocytes necrosis and infarct area	[128, 129]
Platelet	GPIIb-IIIa	Vascular injury	RGD	Imaging the aggravation of platelets.	[130]

HPMA, *N*-(2-hydroxypropyl)methacrylamide; LCCA, ligated left common carotid arteries; MI, myocardial infarction.

### Target ECs in vascular injured sites

Vascular intima, which is composed of a continuous ECs layer, maintains the physiological homeostasis of blood vessels [131]. During cardiovascular events, activated ECs can undergo a dramatic transition in their functional phenotype in response to injury and inflammation [132, 133]. Furthermore, inflammatory ECs highly express many adhesion molecules (e.g. ICAM1, VCAM1, E-selectin and P-selectin), which play an important role in immune recruitment [134, 135]. Thus, designing drugs based on these inflammatory biomarkers of ECs can enhance the likelihood of EC targeting and uptake by activated ECs (Table 2). Our lab’s recent study uses integrin α4β1 highly expressed macrophage membrane to encapsulate rapamycin for inhibiting cell inflammation and autophagy. These biomimetic EVs can significantly target activated ECs (Vcam1^+^) and efficiently reduce inflammation and atherosclerotic plaque [70]. Moreover, MI as coronary acute ischemia and hypoxia-induced injury needs therapeutic angiogenesis to restore the blood supply. Thus, recent study has used cardiac homing peptide conjugated with cardiac stem cell-derived exosomes to target infarcted heart for regenerative therapy. These targeting exosomes can promote EC proliferation, which contributes to promoting angiogenesis and reducing scar size in heart [136].

### Target macrophages/monocytes in vascular injured sites

Macrophages/monocytes, as an important member of immune system, can be recruited to the injured site as soon as 15 min [54], and differentiate into pro-inflammatory M1 and anti-inflammatory M2 macrophages according to the injured degree and type of the injured location [137]; whereas uncontrivable M1 macrophages in cardiovascular will exacerbate a variety of inflammation-based disorders, such as atherosclerosis, MI, intimal hyperplasia and so on [11, 18]. During myocardial I/R or other vascular injured diseases, abnormally accumulating M1 macrophages will result in inflammation and a disturbing reparative stage. Therefore, modulation of macrophage polarization is an important method for myocardial and vascular repair. Recent research has used MSC-derived EVs fusing with platelet membrane to mimic the binding effect of platelet Cd62p to macrophage Psg1. These modified EVs successfully reprogram M1 macrophages to M2 macrophages and accelerate cardiac repair [138]. Moreover, macrophage polarization markers such as M1 marker: MARCO, HLA-DPB1, CD80, CD86; M2 marker: MRC1, CD163, CD209, CLEC7A also can be used to design macrophages’ targeting EVs [139]. Macrophages that engulf excessive lipids and apoptotic cells via scavenger receptors (CD36, MSR1 and LOX-1) will form foam cells in vascular, resulting in tissue inflammation and apoptosis [140]. Nie *et al*. have used KOdiA-PC-modified liposome-like vesicles to target macrophage CD36 receptor and demonstrate that vesicles can co-localize with CD36^+^ macrophages and accumulation in vulnerable and inflammatory atherosclerotic plaque [125].

### Target VSMCs/cardiomyocytes in vascular injured sites

VSMCs are the main cell types involved in constituting arterial vascular wall and ensuring vascular tension. The disorder of VSMCs will trans-differentiate to multiple cells including osteoblasts-like VSMC (Runx2, Msx2), macrophage-like VSMCs (Mac2, Cd11b) and synthetic VSMCs with proliferative property, which contribute to the progression of cardiovascular diseases such as hypertension, atherosclerosis and so on [141]. Thus, recent research mainly focuses on inhibiting the ‘malignant’ proliferation and trans-differentiation of VSMCs in the neointima during vascular injury. Wang *et al*. have used CAR peptide to modify MSC-derived EVs for targeted pulmonary hypertension therapy. CAR-modified EVs significantly target abnormal SMC and inhibit hypoxia-induced proliferation, migration and phenotype transition of SMC during pulmonary hypertension [127]. Moreover, Because PDGFRβ is overexpressed in proliferative SMC, PDGF–BB peptide conjunct dexamethasone–PLGA NP is designed to target injured SMCs, exhibiting significant inhibition of SMCs’ proliferation [126].

Cardiomyocytes as part of muscle cells play an important role in cardiac function. During myocardial I/R injury and infarction, cardiomyocytes as the main stimulus for the inflammation result in excessive necrosis and cardiac dysfunction. Thus, recent researchers have used myocardium‐targeting peptides to modify stem cell-derived exosomes for targeting injured cardiomyocytes, which significantly reduce cardiomyocytes necrosis and infarct area, and have improved cardiac function [128, 129].

## Discussion

EVs, as secreted plasma membrane cargo carriers, are released from diversities of cells into blood flow and substantive organization under both developmental and stress environments [142]. In the past decades, a lot of work have indicated the physiological functions of EVs, which are essential for intercellular communication and the transduction of biological signals [143]. Cardiovascular diseases, which are accompanied by many risk factors, have complex disease processes and pathogenesis. EVs from vascular injured sites, due to their effect on inflammation, thrombosis, angiogenesis and endothelial homeostasis, play an important role in the initiation and progression of vascular regeneration [144–146]. Meanwhile, recent researchers have reported that exosomes derived from MSCs, as stent coating materials, can accelerate re-endothelialization and promote vascular repair after stent implantation [81, 147], whereas there are still three main limitations in EVs’ drug delivery system: (i) the isolation and purification of EVs especially in <100 nm exosomes currently need ultra-high-speed centrifuge with >100 000 g centrifugal force; (ii) given that the content of EV is determined by its donor cell or tissue fluid, the heterogeneity of donor cell is reflected in the EV, which needs bioengineered methods to reduce this heterogeneity; and (iii) the natural EVs, as the cellular membrane leaflet, have low functional proteins to recognize abnormal cells during targeted therapy.

To improve the EV-drug delivery system, researchers utilize a variety of bioengineered methods to modify natural EVs [148]. Recent efforts rationally provide a contemporary design for EVs-secreting cells to increase targeting components of EVs via stimulating induction and biogenetic modulation (Fig. 2). EVs derived from stem cells during inducing conditions (such as inflammation [149, 150], hypoxia [151] and so on) can improve vascular function and reduce unexpected inflammation after injury. Furthermore, EVs also can be pre-loaded with exogenous compounds (such as miRNA [152], lncRNA [153] and cytokines [154]) to enhance the bioactivity of EVs. Meanwhile, recent study has found that overexpression of glycolytic enzyme glyceraldehyde-3-phosphate dehydrogenase in donor cells can increase the loading efficiency of siRNA and the generative rate of EVs [155]. However, compared with recent nanotechnology, the therapeutic effect and targeting accuracy of natural EVs still need to be enhanced.

Nowadays, biomimetic sEVs derived from synthetic cell membranes are widely utilized for targeted drug delivery, which has high biocompatibility, prolonger circulating time as well as specifically targeted ability. Recent research has created newly mimic exosomes, which combine synthetic liposome and macrophage-derived EVs to enhance drug delivery efficiency [156]. Furthermore, our recent researches find that synthetic RBC-derived sEVs can prolong circulating time, significantly attenuate the progression of atherosclerosis [71] and specifically be enriched in low shear stress area [157]. Moreover, synthetic EVs can also be modified to construct targeting structures on the membrane surface via genetic engineering and chemical conjunction [158, 159].

Overall, EVs as cell-based therapies for vascular injury take unique advantages of surviving in the circulation, breaking through the vascular barrier and delivering molecular cargo to recipient cells. Moreover, EVs can be modulated and synthesized through bioengineered methods, which can easily enhance the bioactivity and targeting of EVs. Meanwhile, during vascular injury, EVs as biological agents derived from repairing cells play an important mediator in the pathophysiological process. Bioengineered EVs loading with single or multiple biomolecules and drugs are emerging as therapeutic methods for vascular regeneration.
